# Genome-Wide Identification and Analysis of the NF-Y Gene Family in Potato (*Solanum tuberosum* L.)

**DOI:** 10.3389/fgene.2021.739989

**Published:** 2021-09-17

**Authors:** Zhen Liu, Yuanming Li, Jinyong Zhu, Wenjing Ma, Zhitao Li, Zhenzhen Bi, Chao Sun, Jiangping Bai, Junlian Zhang, Yuhui Liu

**Affiliations:** ^1^State Key Laboratory of Aridland Crop Science, Gansu Agricultural University, Lanzhou, China; ^2^College of Horticulture, Gansu Agricultural University, Lanzhou, China; ^3^College of Agronomy, Gansu Agricultural University, Lanzhou, China

**Keywords:** potato (*Solanum tuberosum*), nuclear factor Y transcription factor, expression profiles, abiotic stress, anthocyanin biosynthesis

## Abstract

Nuclear factor Y (NF-Y) is a ubiquitous transcription factor in eukaryotes, which is composed of three subunits (NF-YA, NF-YB, and NF-YC). NF-Y has been identified as a key regulator of multiple pathways in plants. Although the NF-Y gene family has been identified in many plants, it has not been reported in potato (*Solanum tuberosum*). In the present study, a total of 41 NF-Y proteins in potato (StNF-Ys) were identified, including 10 StNF-YA, 22 StNF-YB, and nine StNF-YC subunits, and their distribution on chromosomes, gene structure, and conserved motif was analyzed. A synteny analysis indicated that 14 and 38 pairs of StNF-Y genes were orthologous to *Arabidopsis* and tomato (*Solanum lycopersicum*), respectively, and these gene pairs evolved under strong purifying selection. In addition, we analyzed the expression profiles of NF-Y genes in different tissues of double haploid (DM) potato, as well as under abiotic stresses and hormone treatments by RNA-seq downloaded from the Potato Genome Sequencing Consortium (PGSC) database. Furthermore, we performed RNA-seq on white, red, and purple tuber skin and flesh of three potato cultivars at the tuber maturation stage to identify genes that might be involved in anthocyanin biosynthesis. These results provide valuable information for improved understanding of StNF-Y gene family and further functional analysis of StNF-Y genes in fruit development, abiotic stress tolerance, and anthocyanin biosynthesis in potato.

## Introduction

Nuclear factor Y (NF-Y) transcription factor, also known as CCAAT-box binding factor (CBF) or heme-associated protein (HAP) (Mantovani, 1999), is a ubiquitous transcription factor in eukaryotes (Zhao et al., 2017). NF-Y is a trimeric transcription factor composed of three distinct subunits, namely, NF-YA, NF-YB, and NF-YC (Nardini et al., 2013). To form the NF-Y complex, the histone folding motifs of NF-YB and NF-YC interact in the cytoplasm to form heterodimer, which is transferred to the nucleus. Upon arrival in the nucleus, the NF-YA subunit is recruited and combines with NF-YB/NF-YC heterodimer to produce mature NF-Y complex (Kahle et al., 2005; Laloum et al., 2013). In mature NF-Y complex, the NF-YA subunit provides a distinctive sequence that specifically binds to the CCAAT *cis*-element and regulates the expression of target genes (Hackenberg et al., 2012; Yan et al., 2013; Cao et al., 2014; Xu et al., 2016). In yeast and mammals, each NF-Y subunit is encoded by a single gene; however, three NF-Y subunits are encoded by multiple genes in plants (Li et al., 1992; Petroni et al., 2012).

In recent years, many NF-Ys were identified in *Arabidopsis* (Riechmann et al., 2000), rice (*Oryza sativa*) (Yang W. et al., 2017), tomato (*Solanum lycopersicum*) (Li et al., 2016), banana (*Musa acuminata*) (Yan et al., 2019), sorghum (*Sorghum bicolor*) (Malviya et al., 2016), watermelon (*Citrullus lanatus*) (Yang J. et al., 2017), and canola (*Brassica napus*) (Liang et al., 2014). And, the functions of many NF-Y genes have been characterized to play multiple roles in plants, including embryogenesis (Kwong et al., 2003), seed germination (Liu et al., 2016), root growth (Sorin et al., 2014), flowering (Cao et al., 2014), fruit ripening (Li et al., 2016), as well as flavonoid biosynthesis (Wang et al., 2020) and response to various abiotic stresses (Li et al., 2008; Ni et al., 2013; Sato et al., 2014; Wang et al., 2018). For example, *AtLEC1* (*AtNF-YB9*) is a key regulator of late embryogenesis and seed development in *Arabidopsis*, and the ectopic expression of *AtLEC1* caused abnormality in tobacco transgenic seedling (Guo et al., 2013). The overexpression of *AtNF-YA1* and *AtNF-YA9* significantly affects male gametophyte development, embryo development, seed morphology, and seed germination (Mu et al., 2013). The overexpression of *AtNF-YA5*, which is strongly induced under drought, increases drought tolerance of *Arabidopsis* plants (Li et al., 2008). The constitutive expression of *TaNF-YA10* significantly increases the sensitivity of *Arabidopsis* plants to salinity (Ma et al., 2015). A recent report suggested that NF-Y complexes composed of NF-YB8, NF-YC1/9, and NF-YA1/9 play an important role in tomato flavonoid biosynthesis, and these complexes are involved in transcriptional regulation and H3K27me3 marking of the *chalcone synthase* (*CHS1*) locus (Wang et al., 2020). Although the NF-Y gene family has been extensively studied in plants, little report is yet available for potato.

Potato, which originated from the Andean regions of Peru and Bolivia (Spooner et al., 2005), is an important food crop worldwide. As a new kind of natural pigment and antioxidant resource, the pigmented potato has attracted more and more attention (Fossen and Andersen, 2000). Flavonoids not only attract insects for pollination but also protect plants against UV-induced damage (He and Giusti, 2010), as well as play a key role in cold and drought stress tolerance (Castellarin et al., 2007; Kim et al., 2017). Furthermore, many flavonoids have strong antioxidant activity and free radical-scavenging ability, which are beneficial to human health, such as preventing cardiovascular disease, controlling obesity, alleviating diabetes, anti-cancer, and so on (Hollman and Katan, 1997; Castañeda-Ovando et al., 2009). However, abiotic stresses can severely restrict the growth and productivity of potato, such as high-temperature stress, cold stress, salinity stress, and drought stress. Given the important role that NF-Y genes play in plants, it is essential to identify and study the NF-Y gene family in the potato genome.

In the present study, we identified a NF-Y gene family with 41 members in potato (*StNFYs*), and comprehensive analyses of the phylogenetic relationships, chromosome distribution, gene structure, sequence features, and gene duplications were further performed. In addition, we analyzed the expression profiles of NF-Y genes in different tissues of double haploid (DM) potato, as well as under abiotic stresses and hormone treatments by RNA-seq downloaded from the Potato Genome Sequencing Consortium (PGSC) database. Furthermore, we performed RNA-seq on white, red, and purple tuber skin and flesh of three potato cultivars at the tuber maturation stage to identify genes that might be involved in anthocyanin biosynthesis. The results could provide a basis for further study on the functional characterization of the NF-Y gene family.

## Materials and Methods

### Identification of StNF-Ys in Potato

The amino acid and nucleotide sequences of potato were downloaded from the PGSC^1^. To identify the potato NF-Y members, two methods were used individually and then combined: (1) the 36 reported *Arabidopsis* NF-Y (AtNF-Y) protein sequences (Riechmann et al., 2000) were downloaded from the *Arabidopsis* information resource (TAIR;^2^). The BLASTP (Altschul et al., 1997) was used to search for StNF-Y members based on 36 AtNF-Y amino acid sequences with an *E*-value ≤ 1e-5. (2) The hidden Markov model (HMM) profiles of the NF-Y domain (PF00808 and PF02045) were downloaded from Pfam^3^. The StNF-Y proteins were identified by HMMER3.1 software^4^. After removing all redundant sequences, these candidate members were submitted to SMART^5^ and NCBI Conserved Domain Data (CDD) to manually screen StNF-Y members for further analysis.

### Sequence Analysis and Structural Characterization

The number of amino acids, theoretical isoelectric point (pl), and molecular weight (MW) were calculated using the ExPasy site^6^ (Gasteiger et al., 2005). The MEME program^7^ was used to identify motifs in StNF-Y sequence with the following parameters: 20 motifs with an optimal motif width of 6–50 amino acid residues and any number of repeats (Bailey et al., 2009). The gene structures of the StNF-Ys were drawn using the Gene Structure Display Server (GSDS 2.0,^8^) (Guo et al., 2007).

### Chromosomal Localization and Gene Duplication

All *StNF-Y* genes were mapped to 12 potato chromosomes based on physical location information from PGSC using MapChart software (Voorrips, 2002). MCScanX (Wang et al., 2012) was used to analyze the duplication events of the *StNF-Y* genes and identified the synteny of *NF-Y* genes between potato and *Arabidopsis*. Ka (non-synonymous) and Ks (synonymous) of each pair of duplicated *NF-Y* genes were calculated using KaKs Calculator 2.0 (Wang et al., 2010).

### Multiple Alignments, Phylogenetic Analysis, and Classification of NF-Ys

The multiple alignments were performed using ClustalW on the 41 StNF-Ys amino acid under default parameters. The full-length amino acid sequences of 41 StNF-Ys, 54 SlNF-Ys (tomato NF-Y), and 36 AtNF-Ys were used for phylogenetic analysis. The Molecular Evolutionary Genetics Analysis (MEGA) 7.0 software (Kumar et al., 2016) was used to construct an unrooted phylogenetic tree with the following parameters: Poisson model and 1,000 bootstrap replications.

### Plant Materials and Treatments

**FIGURE 1:**
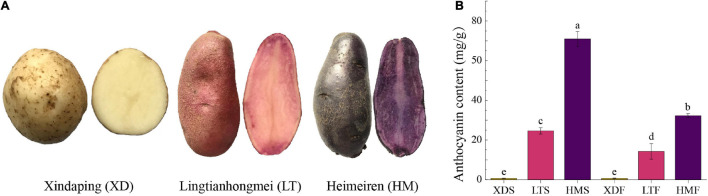
Phenotypes of three potato cultivars in **(A)** “Xindaping” (XD—white skin and white flesh), “Lingtianhongmei” (LT—red skin and red flesh), and “Heimeiren” (HM—purple skin and purple flesh). **(B)** The content of anthocyanin in three potato cultivars. Data represent the mean of three biological replicates ± standard error. Standard errors are shown as bars above the columns. Different letters above bars denote significant difference at *P* < 0.05.

Three potato cultivars, including “Xindaping” (XD—white skin and white flesh), “Lingtianhongmei” (LT—red skin and red flesh), and “Heimeiren” (HM—purple skin and purple flesh), were grown in a greenhouse at Gansu Agricultural University in Lanzhou, Gansu Province, China (Figure 1). Six fresh tubers (diameter = 4–5 cm) were collected from each cultivar for harvesting the skin tissue and flesh tissue. Skin tissues were carefully obtained from the cortical tissue using a scalpel. Flesh tissues were isolated at least 5-mm distance from the skin. The samples were immediately frozen in liquid nitrogen and stored in −80°C refrigerator for further use.

### RNA-Seq Data Analysis

Total RNA of the aforementioned samples was chosen for further RNA-seq library construction. Each sample had three biological replications. Next-generation Illumina sequencing was performed by Sagene Biotech Corporation (Guangzhou, China). The raw RNA-seq data were uploaded to the NCBI (Project ID PRJNA541919). After RNA sequencing, the clean reads were obtained by trimming the raw reads and filtering out contaminants, adapters, Phred scores less than 20, and uncertain bases. The cleaned data were aligned to PGSC_DM_v3.4 gene models downloaded from Solanaceae Genomics Resource at Michigan State University^1^ by Bowtie2 (v2.2.9). Only reads with a perfect match or one mismatch were further analyzed.

### Differential Expression Genes Analysis

Differential genes were identified using fragments per kilobase of exon per million fragments mapped (FPKM). Genes with the absolute value of log_2_ fold change (FC) ≥ 1 and a false discovery rate (FDR) < 0.05 were considered significant differentially expressed genes (DEGs).

The DEGs were annotated against non-redundant database (Nr), SwissProt/UniProt Plant Proteins, Kyoto Encyclopedia of Genes and Genomes (KEGG), Cluster of Orthologous Groups of proteins (COG/KOG), and the potato protein database^9^ by BLASTX, with a cut-off *E*-value of 1e-5. Then, DEGs were subjected to enrichment analysis of GO functions and KEGG pathways.

### RNA Isolation and Quantitative Real-Time PCR

Total RNA extractions were isolated with the RNA extraction kit(Tiangen DP419, Beijing, China). The integrity of RNA was monitored by agarose gel electrophoresis, and the concentration was detected using a NanoDrop ND-2000 spectrophotometer (NanoDrop Technologies, United States). The cDNAs were synthesized using a FastKing RT kit with gDNase (Tiangen KR116, Beijing, China). Quantitative real-time PCR (qPCR) was performed with SuperReal PreMix Plus kit (SYBR Green FP205, Tiangen, Beijing, China) in Bio-Rad CFX96 (Bio-Rad, Hercules, CA, United States). The PCR program was as follows: 30 s at 95°C, followed by 40 cycles of 5 s at 95°C and 30 s at 60°C, followed by 65–95°C melting curve detection. Three independent biological replications were performed. We used the 2^–ΔΔCt^ method (Livak and Schmittgen, 2001) to determine the relative expression levels of genes. The *StEF-1*α (AB061263) was taken as the internal control (Tang et al., 2017). The primers are listed in Supplementary Table 1.

### Expression Pattern Analysis of StNF-Ys in Potato

The expressions of *StNF-Y* genes in 13 tissues (leaves, stamens, shoots, stolons, roots, tubers, carpels, petals, petioles, sepals, flowers, immature fruit, and mature fruit) in DM potato and the whole plant *in vitro* that was treated for abiotic stress (salt treatment: 150 mM NaCl, 24 h; mannitol-induced drought stress: 260 μM mannitol, 24 h; heat treatment: 35°C, 24 h) and hormone treatments [benzylaminopurine (BAP): 10 μM, 24 h; abscisic acid (ABA): 50 μM, 24 h; indole acetic acid (IAA): 10 μM, 24 h; gibberellic acid (GA3): 50 μM, 24 h] were analyzed based on the Illumina RNA-seq data that was downloaded from the PGSC (The Potato Genome Sequencing Consortium et al., 2011). TBtools software was used to draw the heat map (Chen et al., 2018).

The expression patterns of *StNF-Y* genes in three pigmented potato cultivars (XD, LT, and HM) were analyzed based on RNA-seq. The Illumina sequencing was performed by Sagene Biotech Corporation (Guangzhou, China). The raw data were uploaded on NCBI (Project ID PRJNA541919).

### The Interaction Network of NF-Y Proteins

The Search Tool for the Retrieval of Interacting Genes/Proteins (STRING) is a database of known and predicted protein–protein interactions. Based on the RNA-seq dataset of pigmented potato cultivars, we selected StNF-Ys with FPKM > 5 to predict the protein interaction network. The protein interactions were constructed using online STRING v11.0 software^10^ with a combined score > 400 (medium confidence) (Szklarczyk et al., 2016). As active interaction sources, text mining, experiments, databases, co-expression, neighborhood, gene fusion, and co-occurrence were selected. The interaction network was visualized by Cytoscape v3.7.1 (Shannon et al., 2003).

### Statistical Analysis

For qPCR analyses, data were presented as means (±SE) of three biological replicates. Statistical significance was determined by one-way ANOVA followed by the least significant difference (LSD) computed at *P* < 0.05.

## Results

### Identification and Chromosomal Distribution of StNF-Ys

To obtain the NF-Y family members in potato, the HMM and BLASTP algorithm search were combined to analyze the potato genome. A total of 41 *StNF-Y* genes were identified and confirmed in the potato genome, including 10 *StNF-YA*, 22 *StNF-YB*, and 9 *StNF-YC* (Supplementary Table 2). The transcript IDs were converted to corresponding gene IDs and the “SC0003DMG40” in the gene ID was omitted for brevity. The bioinformatics data of StNF-Y members were analyzed, including the number of amino acid residues, theoretical molecular weight (MM), and theoretical isoelectric point (pI). The StNF-Y proteins varied in length and physicochemical properties, the number of amino acid residues of StNF-Y proteins ranged from 120 (PG0013302) to 311 (PG0021365), the molecular weights were between 13.82 kDa (PG0013302) and 33.97 kDa (PG0021365), and the pI values ranged from 4.64 (PG0023065) to 9.65 (PG0002484). The details are listed in Supplementary Table 2.

**FIGURE 2:**
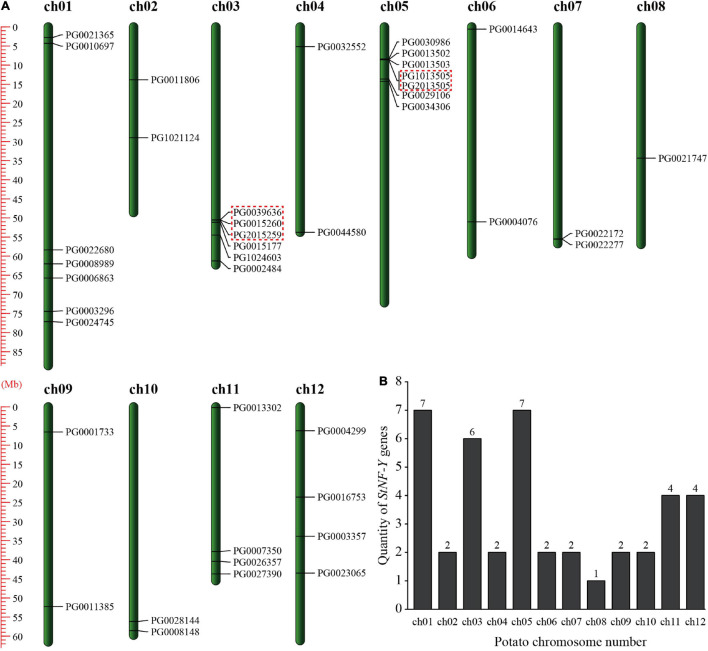
Genomic distributions of 41 *StNF-Y* genes on 12 potato chromosomes. **(A)** “*StNF-Y* gene distribution map” on 12 potato chromosomes. Tandemly duplicated genes were marked by red boxes. **(B)** Numbers of *StNF-Y* genes on each chromosome in potato.

The identified 41 *StNF-Y* genes were distributed unevenly on 12 chromosomes (Figure 2A). Seven *StNF-Y* genes were located on chromosomes 1 and 5, which had the largest number of *StNF-Y* genes. In contrast, only one *STNF-Y* was distributed on chromosome 8, which had the least number of *StNF-Y* genes (Figure 2B). In addition, the high densities of *StNF-Y* genes were distributed at the proximal end of chromosome 1 and the distal end of chromosome 5.

### Multiple Alignments, Phylogenetic Analysis, and Classification of StNF-Ys

**FIGURE 3:**
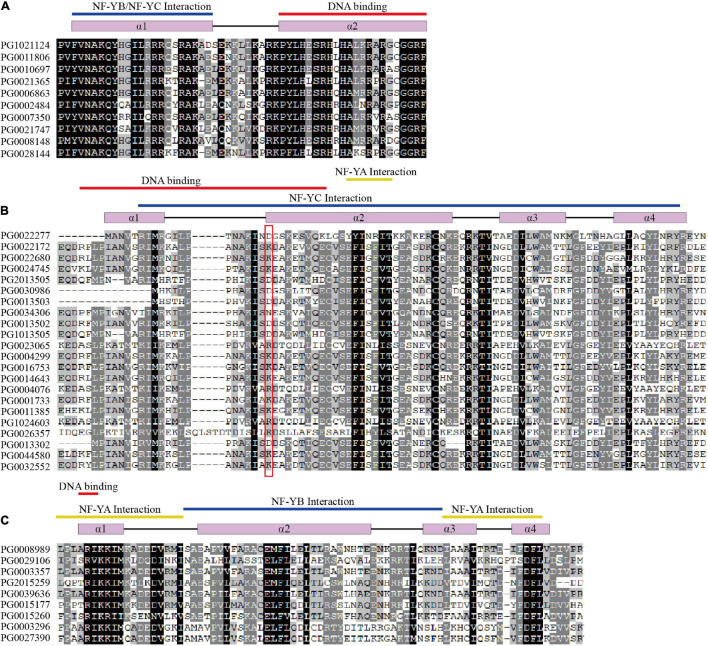
Multiple sequence alignments of StNF-Y family members. **(A)** Multiple alignments of the StNF-YA conserved domains. **(B)** Multiple alignments of the StNF-YB conserved domains. **(C)** Multiple alignments of the StNF-YC conserved domains. The secondary structures of StNF-Ys are indicated at the top of each alignment. The alpha-helices are represented in purple rectangles, and the coils are represented in black lines on the top of the alignment. DNA-binding, yellow lines indicate NF-YA interaction regions, and blue lines indicate NF-YB/YC interaction domains, respectively. The key amino acids that distinguish LEC1 from non-LEC1 are represented in the red box.

Multiple sequence alignments showed that StNF-Y family proteins had conserved region domains as shown in Figure 3. The results showed that each member of StNF-Y contains a heterodimerization domain and a DNA-binding domain that recognizes the CCAAT site. The StNF-YA subunit contained two conserved domains (Figure 3A); one is the NF-YB and NF-YC interaction domain and the other is the DNA-binding domain. In this DNA-binding domain, three histidine (H) and three arginine (R) residues are absolutely conserved and essential (Xing et al., 1993). The conserved domain of StNF-YB subunit was about 93 AAs in length, which contained a NF-YA interaction region, a NF-YC interaction region, and a DNA-binding domain (Figure 3B). Previous studies have shown that the NF-YB subunits could be divided into LECl type and non-LECl type according to sequence similarity; the LECl type is composed of LEC1 and LEC1-like (L1L) (Lee et al., 2003; Siefers et al., 2009).

As shown in Figure 3B, the key amino acids that distinguish Lec1 from non-LEC1 were represented in the red box, and aspartate (D) at this site was considered to be specific for LEC1 type. The conserved domains of StNF-YC subunits contained three heterodimerization regions and one DNA-binding domain, which was about 79 AAs in length (Figure 3C).

**FIGURE 4:**
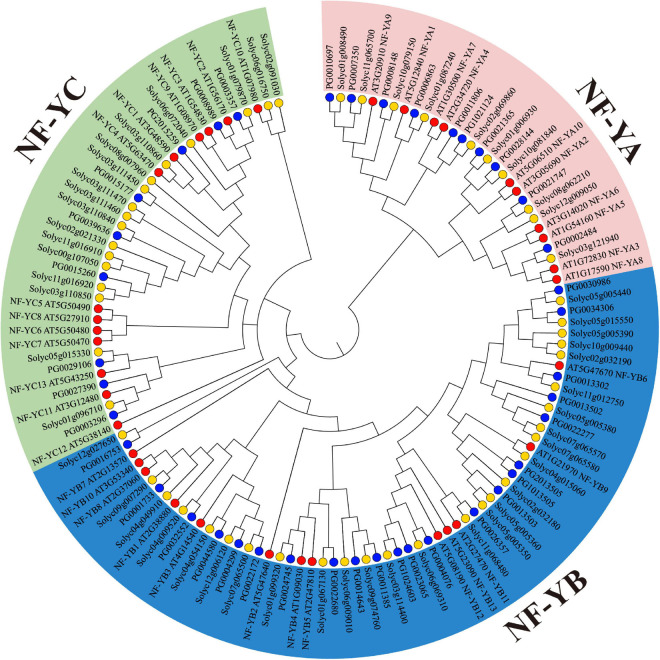
Phylogenetic classification of NF-Ys from potato, tomato, and *Arabidopsis*. The three NF-Y subunits were marked with different colors. The blue circles represent StNF-Ys, the yellow circles represent SlNF-Ys, and the red circles represent AtNF-Ys.

To further investigate the phylogenetic relationships of NF-Ys among potato, tomato, and *Arabidopsis*, an unrooted phylogenetic tree of 41 StNF-Ys, 54 SlNF-Y, and 36 AtNF-Y protein sequences was constructed by MEGA 7.0 software, as shown in Figure 4. The 131 NF-Ys were divided into three distinct subunits (NF-YA, NF-YB, and NF-YC). Ten StNF-Ys, 10 SlNF-Ys, and 10 AtNF-Ys belonged to the NF-YA subunit. Twenty-two StNF-Ys, 27 SlNF-Ys, and 13 AtNF-Ys were assigned to the NF-YB subunit. Nine StNF-Ys, 17 SlNF-Ys, and 13 AtNF-Ys belonged to the NF-YC subunit. Notably, a distinct cluster was identified as LEC1-type group in NF-YB, including AtNF-YB6 (LEC1-like), AtNF-YB9 (LEC1), 13 SlNF-Ys, and eight StNF-YBs (PG1013505, PG2013505, PG0013503, PG0013302, PF0013502, PG0030986, PG0022277, and PG0034306).

### Gene Structure and Conserved Motifs of StNF-Ys

**FIGURE 5:**
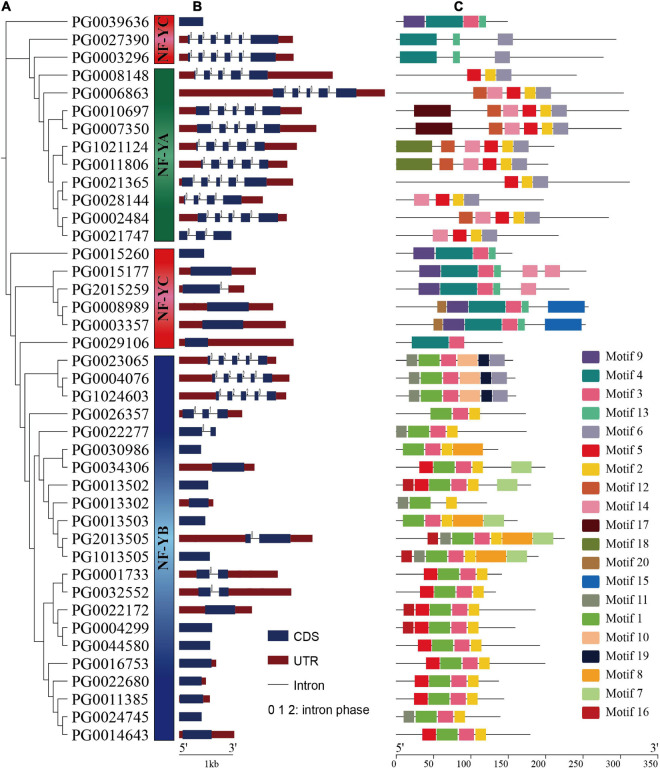
Phylogenetic relationships, gene structure, and conserved motifs analysis of StNF-Ys. **(A)** Phylogenetic tree of StNF-Ys. **(B)** Exon/intron organization of the *StNF-Y* genes. The blue box indicates the exon, and the black line of the same length indicates the intron. The upstream/downstream area is indicated by a red box. The numbers 0, 1, and 2 represent the splicing phase of the intron. **(C)** Distribution of conserved motifs in StNF-Ys. The putative motifs were predicted by MEME program. The 20 boxes of different colors represent 20 different putative motifs. The details of the 20 putative motifs are in Supplementary Table 3.

The full-length amino acid sequences of 41 StNF-Ys were used to construct an unrooted phylogenetic tree by MEGA 7.0 software. Based on phylogenetic analysis of potato, tomato, and Arabidopsis, 41 StNF-Ys were divided into three subunits (Figure 5A). A gene structure analysis can provide insight into the evolution of gene family. Therefore, we analyzed the organization of exons and introns (Figure 5B). The results showed that all of *StNF-YA* genes were separated by introns, of which eight *StNF-YAs* contained four introns, one *StNF-YA* contained three introns, and one *StNF-YA* contained two introns. Most of *StNF-YBs* and *StNF-YCs* did not have introns, including14 *StNF-YBs* and six *StNF-YCs*; the rest of *StNF-YBs* and *StNF-YCs* contained 1–4 introns. In general, the gene structure of *StNF-YA* members was more conserved than that of *StNF-YBs* and *StNF-YCs*.

In order to study the diversification of StNF-Ys, we searched for conserved motifs of these proteins using the online MEME program. The details of the 20 motifs are in Supplementary Table 3. The results showed that the three StNF-Y subunits have a unique motif distribution (Figure 5C). Motifs 12, 17, and 18 were specifically present in the StNF-YA members. Motifs 1, 8, 10, 11, 16, and 19 were unique to the StNF-YB members. Motifs 4, 9, 13, 15, and 20 were present only in StNF-YC members. Motif 2 was distributed in StNF-YA and StNF-YB subunits. Motif 3 was present in StNF-YB and StNF-YC subunits. Motif 6 was widely distributed in three StNF-Y subunits. Overall, each subunit had a conserved motif composition.

### Gene Duplication and Genome Synteny

**FIGURE 6:**
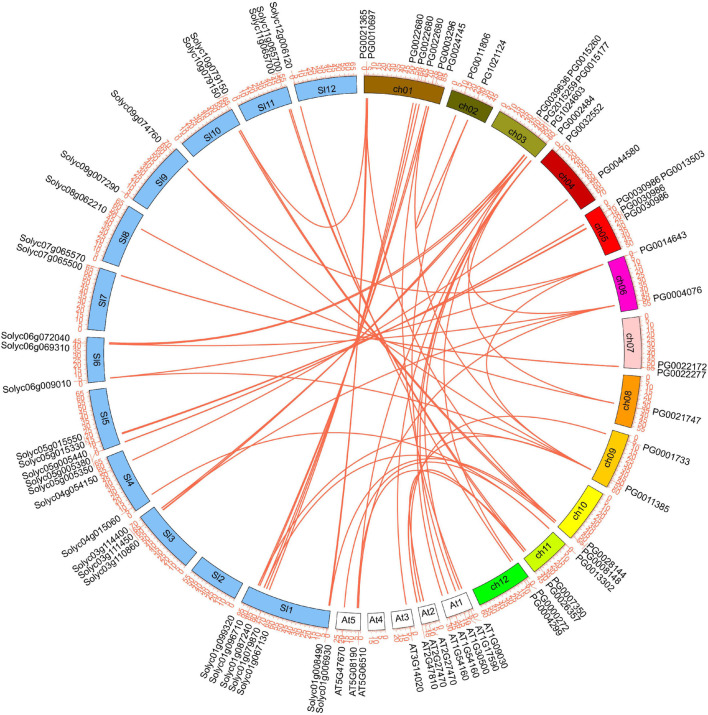
The segmental replication events of *StNF-Y* genes in potato and the orthologous relationships of *StNF-Y* genes with tomato and *Arabidopsis*. Red lines indicate segmental duplication of *StNF-Y* genes and the orthologous relationships of *StNF-Y* genes with tomato and *Arabidopsis*. The chromosome number is indicated at the middle of each chromosome. The white boxes represent *Arabidopsis* chromosome, the blue boxes represent tomato chromosome, and the colored boxes represent potato chromosome, respectively.

Tandem and segmental duplications are crucial for the extension of gene family members and the realization of new functions (Cannon et al., 2004). In the present study, three pairs of *StNF-Y* genes (5/41, 12.20%) were identified as tandemly duplicated genes, of which chromosome 3 had two pairs and chromosome 5 had one pair (Figure 2A and Supplementary Table 4). In addition to tandem duplication, six pairs (10/41, 24.39%) of segmental duplication events were identified (Figure 6 and Supplementary Table 4).

In order to further investigate the potential evolution processes of the NF-Y gene family, we analyzed the synteny relationship of *NF-Ys* among potato, *Arabidopsis*, and tomato. A total of 14 pairs of orthologs were identified between potato and *Arabidopsis*, and 38 pairs of orthologs between potato and tomato were identified (Figure 6 and Supplementary Tables 5, 6).

The substitution rate of non-synonymous (Ka) and synonymous (Ks) is the basis for evaluating the positive selection pressure of duplication events. Ka/Ks = 1 indicates neutral selection, Ka/Ks < 1 denotes purification selection, and Ka/Ks > 1 signifies positive selection. The Ka/Ks of duplication *NF-Y* genes was calculated using KaKs Calculator 2.0. The results showed that the Ka/Ks of tandem and segmental duplications ranged from 0.0787 to 0.9482, with a mean value of 0.3569 (Supplementary Table 4). The Ka/Ks of the orthologous relationship between potato and *Arabidopsis* ranged from 0.0305 to 0.3507, with a mean value of 0.1736 (Supplementary Table 5). And, the Ka/Ks of the orthologous relationship between potato and tomato ranged from 0.001 to 0.9260, with a mean value of 0.2863 (Supplementary Table 6). The Ka/Ks values were < 1, suggesting that these genes had evolved under the effect of purifying selection.

### Expression Profiles of StNF-Y Genes in Different Tissues

**FIGURE 7:**
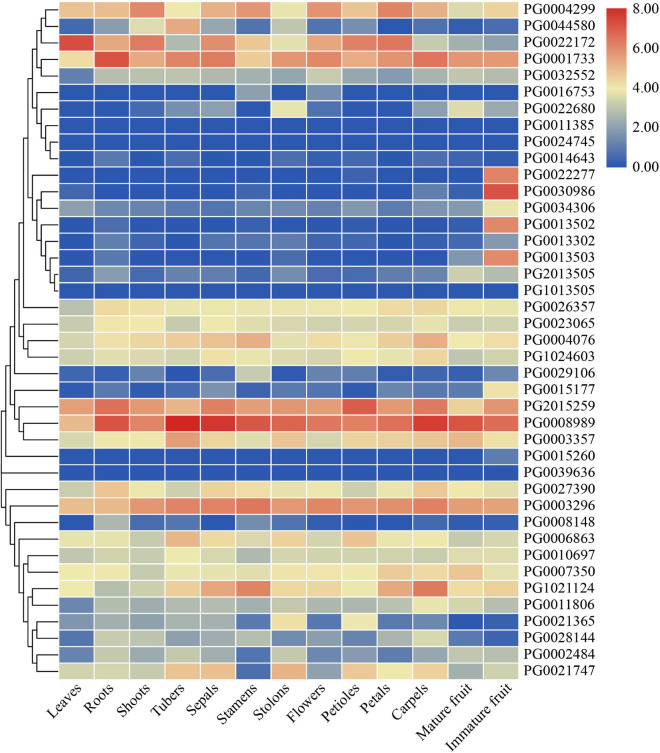
An expression profile analysis of *StNF-Y* genes in different tissues (leaves, roots, shoots, tubers, sepals, stamens, stolons, mature flowers, petioles, petals, carpels, immature fruit, and mature fruit) in double haploid (DM) potato based on the transcriptome data. The color in the heat map represents the fragments per kilobase of exon per million fragments mapped (FPKM) value using logarithm with a base of 2.

Based on transcriptome data downloaded from PGSC, the expression patterns of *StNF-Y* genes in 13 tissues (leaves, roots, shoots, tubers, sepals, stamens, stolons, mature flowers, petioles, petals, carpels, immature fruit, and mature fruit) of DM potato were analyzed (Figure 7 and Supplementary Table 7). The results showed that 14 *StNF-Y* genes (*PG0001733*, *PG0003296*, *PG0003357*, *PG0004076*, *PG0004299*, *PG0006863*, *PG0007350*, *PG0008989*, *PG0010697*, *PG2015259*, *PG1021124*, *PG1024603*, *PG0026357*, and *PG0027390*) were highly expressed in all tissues with FPKM > 5. Four *StNF-Y* genes (*PG0011385*, *PG1013505*, *PG0039636*, and *PG0024745*) were not expressed in all tissues (FPKM = 0). In addition, some StNF-Y genes showed tissue-specific expression patterns, e.g., *PG0021365* was highly expressed in stolon and *PG0044580* was highly expressed in the tuber. It is noteworthy that six *StNF-YB* genes (*PG0013502*, *PG0013503*, *PG0015177*, *PG0022277*, *PG0030986*, and *PG0034306*) were specifically expressed in immature fruit with FPKM > 5 and log_2_FC > 1, which were in the same cluster as *AtNF-YB9* (*LEC1*) and *AtNF-YB6* (*LEC1-like*).

### Expression Profiles of StNF-Y Genes Under Abiotic Stresses and Hormone Treatments

**FIGURE 8:**
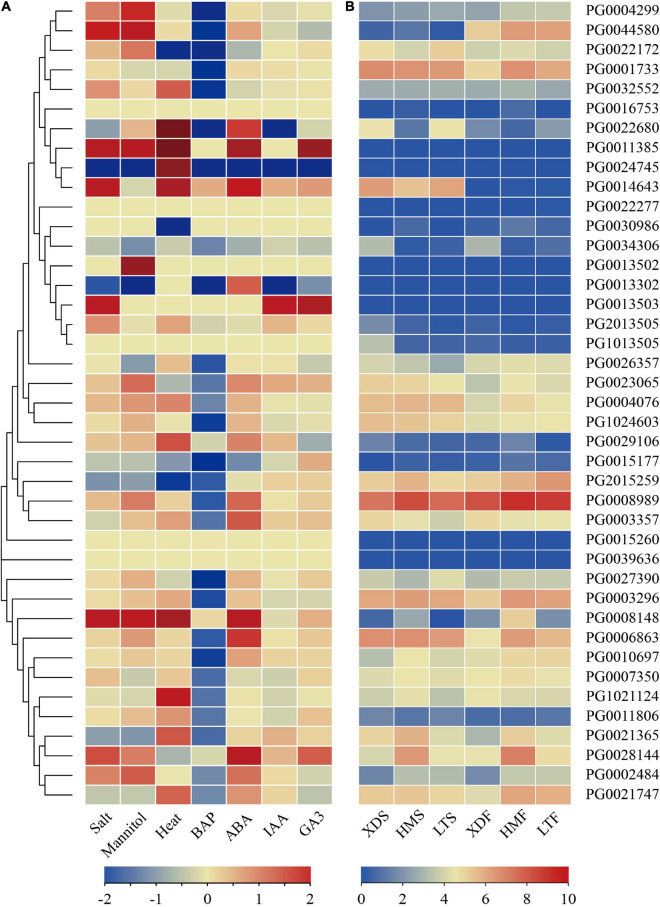
The expression profiles of *StNF-Y* genes. **(A)** The expression profiles of *StNF-Y* genes under abiotic stresses (salt, mannitol, and heat stress) and hormone treatments (BAP, ABA, IAA, and GA3) in DM potato. The color scale was plotted using the log_2_FC of each gene. **(B)** The expression profiles of *StNF-Y* genes in white and pigmented potato tuber skin and flesh. XDS, LTS, HMS represent the white skin of white potato cultivar (Xindaping, XD), the red skin of red potato cultivar (Lingtianhongmei, LT), and the purple skin of purple potato cultivar (Heimeiren, HM), respectively. XDF, LTF, and HMF represent the white flesh of XD, the red flesh of LT, and the purple flesh of HM, respectively. The color in the heat map represents the FPKM value using logarithm with a base of 2. The heatmap was constructed by TBtools software. BAP, benzylaminopurine; ABA, abscisic acid; IAA, indole acetic acid; GA3, gibberellic acid.

The RNA-seq data was downloaded from the PGSC database to analyze the expression profiles of *StNF-Y* genes in DM potato under abiotic stresses. The results showed that six, nine, and 11 *StNF-Y* genes were differentially expressed (FPKM > 1 and | log_2_FC| > 1) under salt, mannitol, and heat treatments, respectively. Among them, four, seven, and nine *StNF-Y* genes were upregulated under salt, mannitol, and heat treatments, respectively. In addition, two *StNF-Y* genes (*PG2015259* and *PG0021365*) were differentially expressed under three stresses, five StNF-Y genes (*PG0002484*, *PG0004299*, *PG0022172*, *PG0028144*, and *PG0044580*) were differentially expressed under two stresses, and 10 genes (*PG0004076*, *PG0008148*, *PG0008989*, *PG0011385*, *PG0014643*, *PG1021124*, *PG0021747*, *PG0022680*, *PG0023065*, and *PG0032552*) were only differentially expressed under single abiotic stress (Figure 8A and Supplementary Table 8).

To further explore the expression changes in the *StNF-Y* genes under various hormone treatments (BAP, ABA, IAA, and GA3), the expression of 41 *StNF-Y* genes was analyzed using the RNA-seq data downloaded from the PGSC database. The results indicated that 22 and 10 *StNF-Y* genes responded to BAP and ABA treatments (FPKM > 1 and | log_2_FC| > 1), respectively. All of 22 *StNF-Y* genes were downregulated under BAP treatments, and all of 10 *StNF-Y* genes were upregulated under ABA treatments (FPKM > 1 and | log_2_FC| > 1). However, all of *StNF-Y* genes were not differentially expressed under IAA and GA3 treatments, implying that *StNF-Y* genes did not respond to IAA and GA3 treatments (Figure 8A and Supplementary Table 8).

Combined with RNA-seq data analysis under abiotic stress and hormone treatment, four *StNF-Y* genes (*PG0002484*, *PG0008989*, *PG0023065*, and *PG0028144*) were differentially expressed (FPKM > 1 and | log_2_FC| > 1) under mannitol and ABA treatments in DM potato. The results indicate that these four *StNF-Y* genes may be involved in drought stress response through an ABA-dependent pathway.

### Expression Profiles of StNF-Y Genes in Pigmented Potato Cultivars

To identify the potential functions of *StNF-Y* genes in flavonoid biosynthesis of potato, the expression patterns of the *StNF-Y* genes in tuber tissues (skin and flesh) of tetraploid pigmented potato cultivars were analyzed according to RNA-seq dataset. The results showed that eight *StNF-Y* genes (*PG0011385*, *PG0013502*, *PG0013503*, *PG0013302*, *PG0015260*, *PG0024745*, *PG0039636*, and *PG0022277*) were not expressed (FPKM = 0) in the skin, and 12 *StNF-Y* genes were differentially expressed in pigmented skin compared with the white skin of XD (FPKM > 1 and | log_2_FC| > 1). Among them, six genes (*PG0002484*, *PG0008148*, *PG0008989*, *PG0010697*, *PG0028144*, and *PG0044580*) were upregulated and four genes (*PG0022680*, *PG0034306*, *PG1013505*, and *PG2013505*) were downregulated in LTS and one gene (*PG0002484*) was upregulated and five genes (*PG0003357*, *PG0026357*, *PG0034306*, *PG1013505*, and *PG2013505*) were downregulated in HMS. In pigmented skin, one gene (*PG0002484*) was upregulated both in the skin of HM and LT, and three genes (*PG0034306*, *PG1013505*, and *PG2013505*) were downregulated both in the skin HM and LT (Figure 8B and Supplementary Table 9).

In tuber flesh, eight *StNF-Y* genes were not expressed (FPKM = 0), and these eight genes also were not expressed in the skin. Compared with the white flesh of XD, a total of 17 *StNF-Y* genes were differentially expressed (FPKM > 1 and | log_2_FC| > 1) in pigmented flesh, of which eight genes (*PG0001733*, *PG0002484*, *PG0003296*, *PG0004299*, *PG0006863*, *PG0021365*, *PG0021747*, and *PG0044580*) were upregulated in both LTF and HMF and one gene (*PG0034306*) was downregulated in pigmented flesh (Figure 8B and Supplementary Table 9). It is noteworthy that one gene (*PG0002484*) was upregulated, and one gene (*PG0034306)* was downregulated in pigmented tissues (skin and flesh).

**FIGURE 9:**
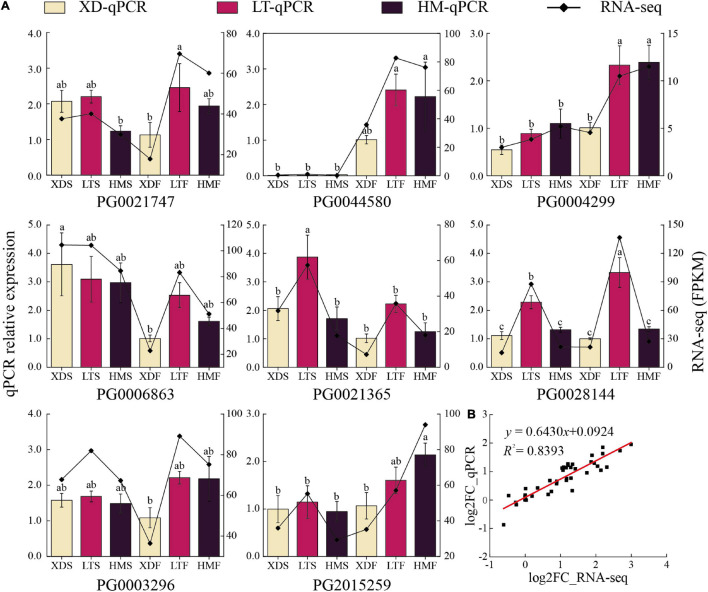
The qPCR expression analyses of eight *StNF-Y* genes in **(A)** the skin/flesh (S/F) of white (XD), purple (HM), and red (LT) cultivars. **(B)** The correlation between qPCR and RNA-seq analysis. Error bars represent the means ± SE of three biological replicates. Different letters above bars denote significant difference at *p* < 0.05. Column graphs represent the relative expression levels of genes determined by qPCR; the values are shown on the left *y*-axis, and line graphs represent the FPKMs obtained by RNA-seq; the values are shown on the right *y*-axis.

Eight *StNF-Y* genes with relatively high expression levels in pigmented tissues were selected as targets, and the reliability of RNA-seq dataset was verified by qPCR. The results confirmed that the qPCR expression patterns were in agreement with the RNA-seq dataset (Figure 9A), showing a high correlation (*R*^2^ = 0.8393) between the RNA-seq dataset and the simple linear regression equation of qPCR (*y* = 0.6430*x* + 0.0924), indicating the consistency of the two analysis methods (Figure 9B).

### The Interaction Network of StNF-Y Proteins

**FIGURE 10:**
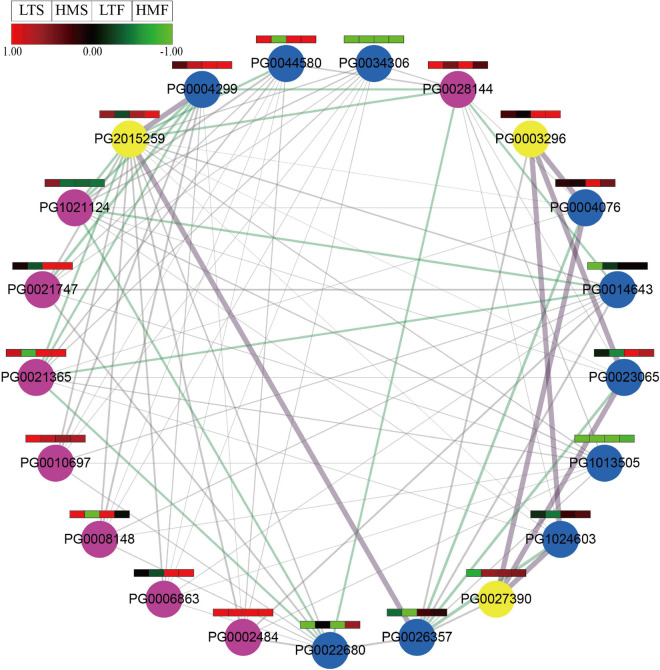
Protein interaction network predicted for StNF-Y (combined score of 400, medium confidence). The purple lines represent the highest confidence (combined score > 900), the green lines indicate high confidence (combined score > 700), and the gray lines indicate medium confidence (combined score > 400). NF-YA subunits are highlighted in purple, NF-YB subunits are highlighted in blue, and NF-YC subunits are highlighted in yellow, respectively. The color scale was plotted using the log_2_FC of each gene.

Previous studies have shown that NF-Y subunits are involved in flavonoid synthesis, and they play a role through interaction with each other (Hackenberg et al., 2012; Mu et al., 2013; Wang et al., 2020). We further investigated the interaction between StNF-Y proteins that might be involved in flavonoid biosynthesis. Based on the RNA-seq dataset of pigmented potato cultivars, we selected highly expressed StNF-Y (FPKM > 5) to predict the protein interaction network with median confidence (combination score > 400). The results showed that a total of 89 pairs of interacting proteins were predicted. Twenty-one StNF-Y proteins (eight StNF-YAs, 10 StNF-YBs, and three StNF-YCs) were included in the protein interaction network, of which, eight StNF-Y genes (*PG0003296*, *PG0004299*, *PG0021365*, *PG0002484*, *PG0006863*, *PG0021747*, *PG0044580*, and *PG0034306*) were differentially expressed in pigmented tissues compared to that in white tissues. These eight StNF-Ys contained four StNF-YA subunits, three StNF-YBs, and one StNF-YC. In addition, we found that StNF-YA subunits did not interact with each other at high confidence (combined score > 400), whereas they showed a direct interaction with StNF-YBs or StNF-YCs (Figure 10 and Supplementary Tables 10, 11).

## Discussion

NF-Ys are important transcription factors in plants, which play critical roles in plant growth, development, and stress responses (Yan et al., 2013; Liu et al., 2016; Xu et al., 2016; Yu et al., 2020). However, little is known about NF-Ys in potato. In the present study, a total of 41 *StNF-Y* genes were identified, including 10 *StNF-YAs*, 22 *StNF-YBs*, and nine *StNF-YCs*. The bioinformatics analysis included a study of the phylogenetic relationships between potato and *Arabidopsis* NF-Y proteins and an analysis of chromosomal location, gene structures, encoded protein conserved motifs, and gene duplication events of *StNF-Y* genes. In addition, the expression pattern of *StNF-Y* genes in different tissues and their response to various abiotic stresses and hormone treatments were analyzed. This study provided comprehensive information for further investigation of their biological functions and the evolution of StNF-Y gene family.

### Expansions of StNF-Y Gene Family in Potato

Gene duplication plays a major role in the expansion of gene families and may be an important driver of evolutionary diversification (Lawton-Rauh, 2003). In the present study, nine pairs of duplication genes were identified in potato, of which the number of *StNF-Y* genes arranged in tandem duplications contributed to 12.20% (5/41) and segmental duplication events contributed to 24.39% (10/41). These results suggested that tandem and segmental duplication events were the main mechanisms of StNF-Y gene family evolution and that some of the *StNF-Y* genes were possibly generated by gene duplication events.

Duplication events often lead to increased functional diversity between duplicated gene pairs, and some new members may acquire new functions or lose their original functions (Dias et al., 2003; Singh et al., 2012). In our study, three *StNF-Y* genes (*PG0039636*, *PG0015260*, and *PG2015259*) were tandem duplication, of which *PG2015259* was downregulated under various abiotic stresses (salt, mannitol, and heat) and highly expressed in 13 tissues (FPKM > 5), while the *PG0039636* and *PG0015260* were not expressed (FPKM = 0), indicating that *PG0039636* and *PG0015260* may become pseudogenes during duplication events and evolution. In addition, some duplication gene pairs were expressed consistently, but some were expressed inconsistently, such as *PG0021747*/*PG0002484* and *PG0021365*/*PG0028144* that were segmental duplication gene pairs. Among them, *PG0021747* and *PG0002484* were upregulated in pigmented flesh; however, *PG0021365* was downregulated and *PG28144* was upregulated under salt and mannitol stresses. In conclusion, gene duplication provided a large number of functional or non-functional genes for the potato genome.

### Phylogenetic Analysis and Evolution of StNF-Ys

To investigate the relationship of NF-Y proteins among potato, tomato, and *Arabidopsis*, an unroot phylogenetic tree was constructed using complete NF-Y protein sequences. The results showed that there were 10 NF-YA in all three plants (potato, tomato, and *Arabidopsis*). However, the amounts of NF-YB and NF-YC varied widely; there are 13 YBs and 13 YCs in *Arabidopsis*, 22 YBs and 9 YCs in potato, and 27 YBs and 17 YCs in tomato, respectively, suggesting that NF-YB and NF-YC play an important role in the expansion of NF-Y gene family.

Studies have shown that AtNF-YC1/2/3/4/9 controls flowering time (Siefers et al., 2009; Kumimoto et al., 2010; Hackenberg et al., 2012). Three StNF-YCs (PG0003357, PG0008989, and PG2015259) were clustered with AtNF-YC1/2/3/4/9, indicating that these StNF-Ys may have a similar function as AtNF-YC1/2/3/4/9. Previous studies have reported that the *AtLEC1* and *AtLEC1-*like play an important role in embryo development and fruit ripening (Kwong et al., 2003; Li et al., 2016; Gago et al., 2017). It is noteworthy that AtLEC1 (AtNF-YB9) and AtLEC1-like (AtNF-YB6) formed a distinct cluster in NF-YB (Figure 4). Our study found that eight *StNF-YB* genes were clustered with *AtLEC1* and *AtLEC1-*like in one cluster, of which six *StNF-YB* genes were specifically expressed in immature fruits. It is suggesting that *LEC1* and *LEC1-*like genes were extensively extended during potato evolution and that these genes may be associated with embryo development and fruit ripening.

### Expression Profile Analysis of StNF-Y Genes Under Abiotic Stress and Hormone Treatment

Previous studies have shown that the NF-Y family is involved in various abiotic stresses (Li L. et al., 2013; Li Y. J. et al., 2013; Alam et al., 2015; Feng et al., 2015). In *Arabidopsis*, *AtNF-YA1* (*At5G12840*) is significantly induced by NaCl, mannitol, PEG, and ABA, and it is involved in the regulation of post-germination growth arrest under salt stress (Li Y. J. et al., 2013). *PG0006863* was homologous to *AtNF-YA1*, and it was highly expressed under mannitol treatment and upregulated under ABA treatment in DM potato, indicating that *PG0006863* may function in a similar way as *AtNF-YA1* under mannitol treatment. Li et al. (2008) found that overexpression of *AtNF-YA5* (*AT1G54160*) in *Arabidopsis* can increase the tolerance of plants to drought stress through the ABA-dependent pathway. *PG0002484* was homologous to *AT1G54160*, which was upregulated under salt, mannitol, and ABA treatments in DM potato, suggesting that *PG0002484* may have similar functions to *AtNF-YA5*. Overexpression of *AtNF-YA7* (*AT1G30500*) enhanced tolerance to various abiotic stresses in *Arabidopsis* (Leyva-González et al., 2012). *PG1021124*, homologous to *AtNF-YA7*, was upregulated under heat stress and differentially expressed under various hormone treatments in DM potato, suggesting that *PG1021124* may have similar functions to *AtNF-YA7* under abiotic stresses. Overexpression of *NF-YA10* can improve tolerance to drought and salt stress in *Arabidopsis* (Ma et al., 2015; Yu et al., 2020). *PG0028144* and *PG0021365* were a pair of segmental duplication genes, which were clustered with *AtNF-YA10* (*AT6G06510*). *PG0021365* was differentially expressed under mannitol and heat treatments, while *PG0028144* was upregulated under salt, mannitol, and ABA treatments. The results indicated that *PG0028144* may be induced by drought stress in an ABA-dependent manner, while *PG0021365* was not. Sato et al. (2019) found that overexpression of *AtNF-YB2* (*AT5G47640*) and *AtNF-YB3* (*AT4G14540*) specifically enhances drought and heat stress tolerance, respectively. *PG0004299*, *PG0044580*, and *PG0022172* were clustered with *AtNF-YB2* and *AtNF-YB3* in one cluster. Among them, *PG0004299* and *PG0044580* had the same expression pattern, which were upregulated under salt and mannitol treatments, indicating that *PG0004299* and *PG0044580* may have similar functions to *AtNF-YB2*. The expression of *PG0022172* was upregulated under mannitol treatment, and downregulated under heat treatment, suggesting that *PG0022172* may have functions of both *AtNF-YB2* and *AtNF-YB3*. In addition, *PG0008989* and *PG0023065* were upregulated under mannitol and ABA treatments, while *PG2015259* was downregulated under three abiotic stresses (salt, mannitol, and heat), suggesting that these *StNF-Y* genes might be putative regulators of response to abiotic stress.

### Expression of StNF-Y Genes in Pigmented Tuber Tissue

The expression profiles of *StNF-Y* genes in different tuber tissues (skin and flesh) of tetraploid pigmented potato cultivars were analyzed based on the RNA-seq dataset. Studies have shown that the NF-Y genes play crucial roles in flavonoid biosynthesis, e.g., NF-YB8 interacts with NF-YC1 and then recruits NF-YA1/9 to form NF-Y complexes that bind the CCAAT element in the CHS1 promoter to regulate the flavonoid biosynthesis in tomato (Wang et al., 2020). *PG0001733* was homologous to *AtNF-YB8* (*AT2G37060*) and clustered with SlNF-YB8c in one cluster, which was upregulated in pigmented flesh (LTF and HMF). *PG2015259* was clustered with AtNF-YC1 (AT3G48590) and SlNF-YC1a (Solyc03g110860) in one cluster and was highly expressed in LTF and upregulated in HMF. *PG0006863* and *PG0008148* were homologous to *SlNF-YA9* (*Solyc01g087240*), of which *PG0006863* was upregulated in LTF and HMF and *PG0008148* was upregulated in LTS and LTF. In addition, *PG0010697* was homologous to *SlNF-YA1a* (*Solyc01g008490*) and was highly expressed in pigmented tissues and upregulated in LTS. These results indicated that these five genes may have similar functions to *SlNF-YA1/9*, *SlNF-YB8c*, and *SlNF-YC1*a in flavonoid biosynthesis. Furthermore, six genes were upregulated in pigmented tissues (LTF and HMF), of which three genes (*PG0002484*, *PG0021365*, and *PG0021747*) belonged to StNF-YA subunit, two genes (*PG0004299* and *PG0044580*) were assigned to StNF-YB subunit, and one gene (*PG0003296*) was grouped into StNF-YC subunit.

In addition, we analyzed the correlation coefficient (CC) between differentially expressed *StNF-Ys* of potato tuber skin and flesh in RNA-seq data and anthocyanin content. The results showed that the expression of some *StNF-Y* genes was highly correlated to anthocyanin content in tuber flesh, such as *PG0044580* and *PG0021747* with CC > 0.7; *PG0004299*, *PG0002484*, and *PG0010697* with CC > 0.8; and *PG2015259* (CC = 0.8) (Supplementary Table 12), suggesting that these *StNF-Y* genes may be involved in the anthocyanin biosynthesis in potato tuber flesh, and their functions require further investigation.

Through the prediction of StNF-Y protein interaction network, we found that there were interaction relationships (high confidence with combined score > 700) between these differentially expressed genes (Figure 10 and Supplementary Table 10), i.e., two StNF-YAs (PG0021747 and PG0021365) directly interacted with one StNF-YB (PG0004299), and one StNF-YB (PG0004299) and one StNF-YA (PG0021365) interacted with one StNF-YC (PG2021259). These StNF-Ys may be involved in the biosynthesis of anthocyanins by interacting with each other to form the completed NF-Y complex, and their functions need to be further studied.

## Conclusion

In this study, we identified a total of 41 *StNF-Y* genes from the potato genome, including 10 *StNF-YA*, 22 *StNF-YB*, and nine *StNF-YC*, and their distribution on chromosomes, multiple alignments, gene structure, conserved motifs, and phylogenetic relationships were analyzed. Synteny analysis showed that nine pairs of duplication genes were identified in the potato genome, which played an important role in the expansion of the StNF-Y gene family. Synteny analysis indicated that 14 and 38 pairs of StNF-Y genes were orthologous to *Arabidopsis* and tomato, respectively. Six *StNF-YB* genes were specifically expressed in immature fruit, which may be involved in fruit ripening. In addition, the *StNF-Y* genes displayed different expression patterns under the salt, mannitol, heat, and hormone treatments in DM potato. Furthermore, we analyzed the expression of *StNF-Y* genes in tetraploid pigmented potato cultivars and the protein interaction networks that were highly expressed in anthocyanin biosynthesis. The results of this study provide valuable information for further studies on the functions of the StNF-Y gene family.

## Data Availability Statement

The original contributions presented in the study are included in the article/Supplementary Material, further inquiries can be directed to the corresponding author/s.

## Author Contributions

YHL, JB, and JLZ conceptualized the study. ZL, YML, JYZ, WM, ZB, and ZTL curated the data. ZL and CS conducted the formal analysis. ZL, YHL, and JLZ acquired funding. ZL contributed the software and wrote the original draft. YHL wrote, reviewed, and edited the manuscript. All authors contributed to the article and approved the submitted version.

## Conflict of Interest

The authors declare that the research was conducted in the absence of any commercial or financial relationships that could be construed as a potential conflict of interest.

## Publisher’s Note

All claims expressed in this article are solely those of the authors and do not necessarily represent those of their affiliated organizations, or those of the publisher, the editors and the reviewers. Any product that may be evaluated in this article, or claim that may be made by its manufacturer, is not guaranteed or endorsed by the publisher.

## Footnotes

1
http://solanaceae.plantbiology.msu.edu/pgsc_download.shtml

2
http://www.Arabidopsis.org

3
http://pfam.xfam.org

4
http://hmmer.org/download.html

5
http://smart.embl-heidelberg.de

6
http://web.expasy.org/protparam

7
http://alternate.meme-suite.org/tools/meme

8
http://gsds.cbi.pku.edu.cn

9
ftp://ftp.jgi-psf.org/pub/compgen/phytozome/v9.0/Stuberosum

10
http://string-db.org/

## Supplementary Material

Supplementary Table 1 Primers used for qPCR.

Supplementary Table 2 Characteristic features of 41 StNF-Y genes.

Supplementary Table 3 Conserved motifs identified by MEME among StNF-Ys.

Supplementary Table 4 Ka/Ks ratios of tandemly and segmentally duplicated *StNF-Y* genes.

Supplementary Table 5 Ka/Ks ratios of the syntenic relationships for NF-Y genes between potato and *Arabidopsis*.

Supplementary Table 6 Ka/Ks ratios of the syntenic relationships for NF-Y genes between potato and tomato.

Supplementary Table 7 RNA-seq data of StNF-Y genes in different tissues.

Supplementary Table 8 RNA-seq data of StNF-Y genes under abiotic stress and hormone treatment.

Supplementary Table 9 The FPKM value of StNF-Y genes in white and pigmented tissues.

Supplementary Table 10 Protein interaction network of StNF-Ys.

Supplementary Table 11 The FPKM value of genes in protein interaction networks.

Supplementary Table 12 The correlation between the expression of StNF-Ys and anthocyanin content in potato tuber (skin and flesh).

## Abbreviations

StNF-Y: nuclear factor Y transcription factor in potato
DM: double monoploid potato
HMM: hidden Markov model
Ka: non-synonymous substitution rate
Ks: synonymous substitution rate
PG: PGSC0003DMG40
qPCR: quantitative real-time PCR
CC: correlation coefficient.
